# Case report: Five patients with myocarditis after mRNA COVID-19 vaccination

**DOI:** 10.3389/fped.2022.977476

**Published:** 2022-08-18

**Authors:** Hiroki Murase, Yiqing Zhu, Keiya Sakaida, Hayato Mizuno, Hiromitsu Mori, Hideyuki Iwayama, Noriyuki Suzuki, Noriko Nagai, Akihisa Okumura

**Affiliations:** ^1^Department of Pediatrics, Okazaki City Hospital, Okazaki, Japan; ^2^Department of Pediatrics, Aichi Medical University School of Medicine, Nagakute, Japan; ^3^Department of Cardiology, Okazaki City Hospital, Okazaki, Japan

**Keywords:** troponin I, diagnosis, myocarditis, mRNA COVID-19 vaccination, electrocardiogram

## Abstract

**Objectives:**

To describe clinical features and laboratory data of myocarditis after the mRNA COVID-19 vaccine in children.

**Methods:**

We reviewed patients younger than 18 years of age, who visited our hospital because of myocarditis within 1 week after BNT162b2 from June 2021 to January 2022.

**Results:**

We identified five male patients aged 12–16 years who presented to our hospital with myocarditis within 2–3 days after the second dose of BNT162b2 COVID-19 vaccination between June 2021 and January 2022. All patients experienced chest pain, and fever, pain other than chest pain, and shortness of breath were present in two, three, and two patients, respectively. The serum troponin I level was increased in all patients except one, and electrocardiogram (ECG) showed ST elevation in all patients. Echocardiography revealed pericardial effusion and decreased ejection fraction in three and one patients, respectively. In accordance with the Japanese guidelines for myocarditis, the patients were treated with colchicine and aspirin. Chest pain improved within a few days with no hemodynamic instability. The patients were discharged with no sequelae.

**Conclusions:**

ST changes on ECG and elevated troponin I levels may aid the diagnosis of myocarditis after mRNA COVID-19 vaccination

## Introduction

Vaccines are the mainstay intervention for controlling the SARS-CoV-2 pandemic. In Japan, the messenger RNA (mRNA) coronavirus disease 2019 (COVID-19) vaccine was approved for children aged ≥12 years in June 2021. Although the BNT162b2 vaccine is effective for children aged 12–15 years (1, 2), myocarditis is a potential adverse effect (3). In Israel, myocarditis occurred in 1 in 20,000 persons aged 18–30 years and was more common in males than females (4). The Advisory Committee on Immunization Practices in United States reported that the myocarditis risk after mRNA vaccination was 10-fold higher among males aged <30 years compared to those aged >30 years; approximately 66.7 per 1 million males aged 12–17 years developed myocarditis after the second vaccine dose (5). In the United States Vaccine Adverse Event Reporting System, there were 660 patients with myocarditis younger than age 18 years fulfilling the definition of the Centers for Disease Control and Prevention until July 7, 2022 (6). The European Medicines Agency reported 145 cases of myocarditis and 138 cases of pericarditis out of 177 million doses of the BNT162b2 vaccine, and 9 cases of myocarditis and 19 cases of pericarditis out of 20 million doses of the mRNA-1273 vaccine (7).

In Japan, the Ministry of Health, Labour and Welfare (MHLW) reported that myocarditis occurred in 3.7 per 1 million males aged 12–19 years after BNT162b2 vaccination (8). Some cases of myocarditis after COVID19 vaccination have been reported in the Japanese population, but there are few reports among teenagers (9–11). The actual state of myocarditis after BNT162b2 vaccine is not completely clear. We identified five patients with myocarditis after BNT162b2 vaccination, and herein report their clinical manifestations and laboratory data.

## Methods

Okazaki City Hospital is a tertiary emergency hospital that provides services to Okazaki City and Koda Town, which contain approximately 30,000 children aged 12–19 years. In this region, a COVID-19 vaccination program for children aged ≥12 years was initiated in June 2021 using the BNT162b2 vaccine. Children who developed myocarditis after vaccination would be expected to visit Okazaki City Hospital. Waiver of informed consent was approved by institutional review board of Okazaki City Hospital, because we retrospectively analyzed the existing data with no identifiable private information.

This study is a retrospective case series including patients aged <17 years who presented to Okazaki City Hospital with myocarditis within 1 week after COVID-19 vaccination, between June 2021 and January 2022. We extracted clinical information from the medical records of hospitalized patients. In this study, myocarditis was diagnosed when a patient with one or more clinical symptoms including chest pain, pressure, discomfort, dyspnea, shortness of breath, pain during breathing, palpitations, and syncope had at least one or more of the following pathological findings: (1) elevated serum troponin level, (2) electrocardiogram (ECG) abnormalities consistent with myocarditis, (3) echocardiogram abnormalities such as reduced ejection fraction and wall motion abnormalities. Patients were excluded when there were other identifiable underlying conditions causing cardiac dysfunction. These conditions met are the criteria for probable myocarditis proposed by Gargano et al. (12). All patients fulfilled the Brighton Collaboration definition for an adverse reaction to immunization (13).

The following information was collected from the patients' medical records: age, sex, presence or absence of chest pain, fever, shortness of breath, fatigue, pain other than chest pain, length of hospitalization, and treatment. We also recorded the serum C-reactive protein and troponin I levels (reference level: <28.6 pg/mL), and ECG and echocardiographic findings. The initial echocardiogram findings were recorded by the attending pediatrician and subsequently confirmed by a board-certified pediatric cardiologist within 2 days. In this study, ejection fraction of 50% or less was defined as reduced ejection fraction. Cardiac MRI with gadolinium enhancement was performed only in one patient, because abnormal findings were considered unlikely in most patients, judging from echocardiographic findings. Quantitative antigen test for SARS-CoV-2 was performed using nasopharyngeal swab in all patients. Systematic viral screening was not performed, although we asked the presence or absence of other prodromes such as viral infections.

## Results

We identified five patients with myocarditis after mRNA COVID-19 vaccination during the study period (Table 1). The median age of those patients was 14 years (range: 12–16 years) and they were all male. The patients developed chest pain a median of 3 days (range: 2–3 days) after the second vaccination. The chest pain was described as a feeling of tightness in the anterior chest that lasted for several hours. Clinical symptoms other than chest pain included fever, pain other than chest pain, and shortness of breath in 2, 3, and 2 patients, respectively. Quantitative antigen test for SARS-CoV-2 was negative in all patients.

**Table 1:** Clinical manifestations.

**Pt**	**Age (yrs)** ** sex**	**Number of vaccinations**	**Interval from vaccination to onset (days)**	**Chest pain**		**Fever**	**Pain other than chest**	**Palpitations or shortness of breath**	**Fatigue**	**Herat rate (bpm)**	**Blood pressure (mmHg)**
				**Location**	**Duration (hours)**						
1	16 male	2	3	Left anterior chest	0.5	Yes	Tooth	No	No	71	115/61
2	15 male	2	3	Anterior chest	A few minutes	Yes	Head, neck, and back	Yes	No	73	104/65
3	14 male	2	2	Anterior chest	1.5	No	No	No	Yes	76	141/84
4	12 male	2	3	Left anterior chest	0.5	No	Head	Yes	No	66	132/78
5	12 male	2	2	Anterior chest	0.5	No	No	No	Yes	82	102/57

Pt, Patient.

**Figure 1:**
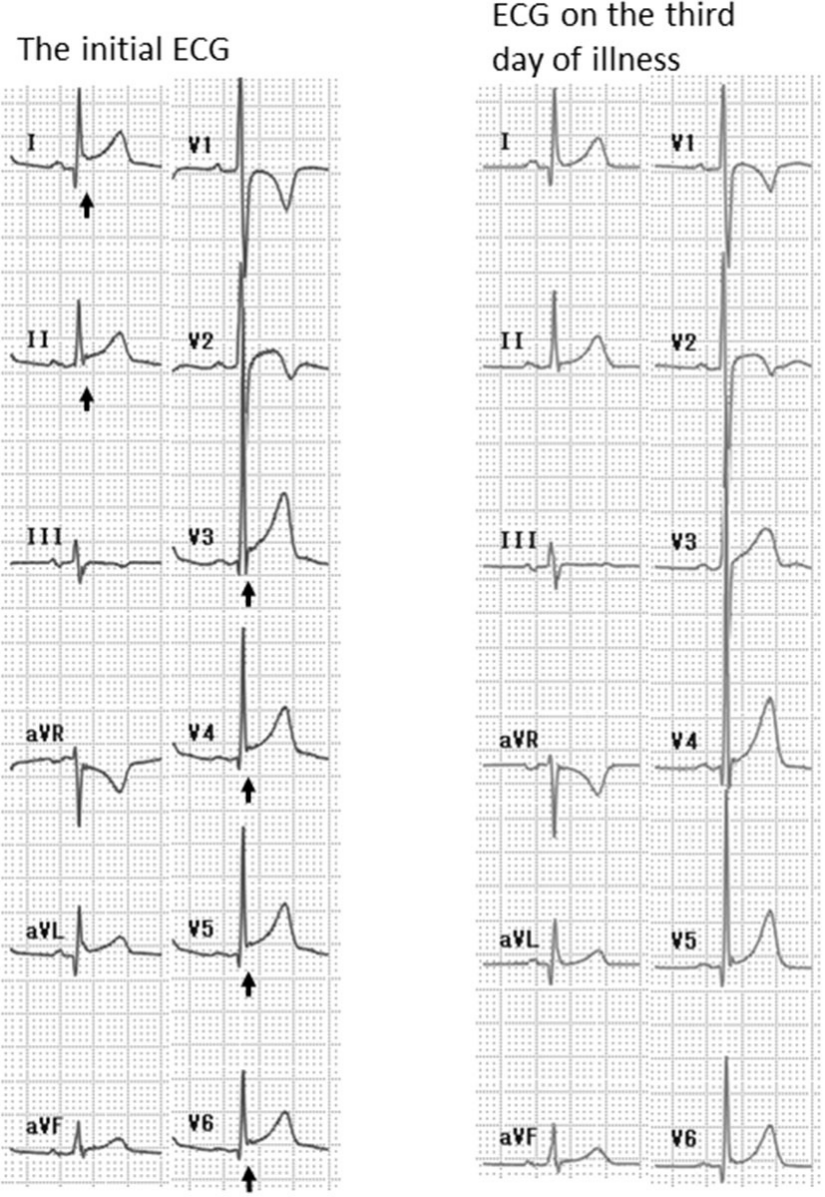
Electrocardiogram (ECG) of Patient 5. The initial ECG showed ST-segment elevation in leads I, II, and V3–6 (arrows). The ECG on the third day of illness was normal.

During the clinical course, an increased serum troponin I level was observed in all patients except one (Table 2). In the remaining patient, the serum troponin I level was at the upper limit of the reference value (reference level: <28.6 pg/mL). In most patients, troponin I reached a peak value within 3 days after admission, and normalized in all cases by 3 months later (Table 2). N-terminal pro-brain natriuretic peptide (NT-proBNP) was measured in 3 of 5 patients. It was elevated in one patient (261 pg/mL, reference level: <125 pg/mL), in whom troponin I was markedly elevated (Table 2). All patients had ST elevation on ECG (Figure 1) during clinical course. In Patients 1 and 5, ST elevation and a markedly elevated serum troponin I level were present at the time of presentation. Patients 2 and 4 had relatively mild ST and serum troponin I elevation at admission. In Patient 3, ST-segment changes were not present at the time of presentation, but appeared 4 h later. In this patient, the serum troponin I level was within the normal range (Troponin I: 24.7 pg/mL) at the time of presentation and reached the upper limit of the normal range 4 days later (Troponin I: 28.5 pg/mL). Continuous ECG monitoring was performed in all patients, and no atrial or ventricular arrhythmias had been noticed.

**Table 2:** Electrocardiogram, echocardiogram, laboratory data.

**Pt**	**ST elevation on ECG**		**Echocardiogram**			Serum troponin I level (pg/mL)*			**NT-proBNP level (pg/mL)^†^**	**Treatment**	**Other drugs**	**Duration of hospitalization (days)**	**Outcome**
	**Initial ECG**	**During clinical course**	**Ejection fraction**	**Wall motion**	**Pericardial effusion**	**Initial value**	**Peak value**	**Normalization**					
1	I,II,III, aVL, aVF, V2-6	Normal (4th day of illness)	65%	Normal	Yes	13,237	16,818 (2nd day of illness)	34.6 (1 month later)	NA	Colchicine	Loxoprofen, omeprazole	8	Full recovery
2	V1,2	Normal (2nd day of illness)	45%	Normal	No	33	33 (1st day of illness)	27.1 (2nd day of illness)	11	ASA	Acetaminophen, famotidine	3	Full recovery
3	V2-V4	V2-6 elevation (4 h later) normal (2nd day of illness)	55%	Normal	No	24.7	28.5 (3rd day of illness)	27.9 (1 month later)	9	ASA	Acetaminophen, famotidine	3	Full recovery
4	V1-V2	Normal (15th day of illness)	63%	Normal	Yes	31.1	40.6 (21th day of illness)	21.0 (3 months later)	NA	Colchicine, ASA	Acetaminophen, famotidine	0	Full recovery
5	I,II,V3-6	Normal (4th day of illness)	70%	Normal	Yes	1,369	1,369 (1st day of illness)	20.4 (1 month later)	261	ASA	Acetaminophen, famotidine	5	Full recovery

Pt, Patient; ECG, electrocardiogram; NT-proBNP, N-terminal pro-brain natriuretic peptide; NA, not assessed; ASA, aspirin.

*Reference level: <28.6 pg/mL.

^†^Reference level: <125 pg/mL.

At presentation, echocardiography showed small amount of pericardial effusion in three patients and slightly decreased ejection fraction in one patient. Cardiac MRI was performed in Patient 5 and showed no abnormal findings.

According to Japanese guidelines for myocarditis, patients were treated with colchicine, aspirin, acetaminophen, loxoprofen, and famotidine. Chest pain improved within a few days, and none of the patients were hemodynamically unstable. The median duration of admission was 3 days (range: 0–8 days), and all patients were discharged with no sequelae.

### Representative case

Patient 5 was a 12-year-old boy who presented with chest pain. His personal and family history were unremarkable. After the first dose of BNT162b2 vaccine, the patient experienced chest pain that lasted for several hours and resolved spontaneously. Two days before presentation, the patient received the second dose of BNT162b2 vaccine. One day after vaccination, he developed fever and fatigue, followed by chest pain on the day of presentation. At presentation, the patient's body temperature was 37.0°C, the heart rate was 82 beats/min, blood pressure was 102/57 mmHg, and the respiratory rate was 18 breaths/min. Physical examination was unremarkable. Laboratory tests revealed a white blood cell count of 8,700/μL, creatine kinase of 278 U/L (reference level: <248 U/L), troponin I of 1,369 pg/mL, and N-terminal pro-brain natriuretic peptide (NT-proBNP) of 261 pg/mL (reference level: <125 pg/mL). The ECG showed ST elevation in leads I, II, and V3–6 (Figure 1). Echocardiography revealed a left ventricular ejection fraction of 68%, mild pericardial effusion, and increased epicardial echodensity. A diagnosis of myocarditis after mRNA COVID-19 vaccination was made, and the patient was treated with 30 mg/kg of aspirin and famotidine. On the next day, the chest pain improved, and the troponin I and NT-proBNP levels decreased to 820.4 and 74 pg/mL, respectively. On the fourth day of admission, the chest pain resolved, the troponin I level decreased to 355 pg/mL, ECG was normalized, and cardiac contrast-enhanced magnetic resonance imaging revealed no delayed excretion of contrast medium. The patient was discharged on the fifth day.

## Discussion

In the study region, a total of 22,557 individuals aged 12–19 years had received two doses of BNT162b2 by January 26, 2022 (14). Assuming that all children with myocarditis after mRNA COVID-19 vaccination presented to our hospital, the proportion of individuals with myocarditis was estimated to be 0.022%. The MHLW reported that ~3.7 per million (0.00037%) males aged 12–19 years developed myocarditis and pericarditis after BNT162b2 vaccination (8), which is a markedly lower rate than in the present study. This discrepancy may be because some patients with myocarditis were missed, because mildly affected patients may not have visited a hospital in the MHLW study. In addition, increased public and physician awareness of the adverse effects of vaccination may have resulted in improved detection of mildly affected patients with myocarditis in the present study.

Mevorach et al. (4) reported that myocarditis occurred in 0.015% of individuals after two doses of mRNA COVID-19 vaccine. Several studies have reported that myocarditis mostly occurs after the second dose of mRNA COVID-19 vaccine (12, 15–17). Therefore, myocarditis after mRNA COVID-19 vaccination is presumed to be more frequent after the second than first dose. In the present study, all of the patients were male, in line with previous studies (12, 15–17); this suggests that males are at an increased risk of myocarditis after mRNA COVID-19 vaccination.

The median interval from the second vaccination dose to the onset of myocarditis was 3 days in this study. Oster et al. (17) reported a median interval from vaccination to myocarditis of 3 days after the first vaccination and 2 days after the second vaccination. Similarly, Truong et al. (18) reported that myocarditis developed a median of 2 days after the second vaccination. Several other studies also reported an interval of 2–3 days between vaccination and myocarditis (12, 15, 16, 19).

In the present study, chest pain was observed in all patients, and fever, shortness of breath, and pain other than chest pain were also common. Marshall et al. (16) reported chest pain in all patients, fever in 28%, pain (including myalgia and abdominal pain) in 85%, and fatigue in 43%. Other studies have also reported high rates of chest pain (89–100%), fever (24–67%), dyspnea (12–40%), and pain other than chest pain (12–85%) (15–17, 19). Therefore, chest pain is almost always present in children with myocarditis after mRNA COVID-19 vaccination; individuals receiving an mRNA COVID-19 vaccine should be instructed to seek medical attention in case of chest pain.

In the present study, ST elevation was present in all patients with myocarditis. In the study of Marshall et al., all seven patients with myocarditis after mRNA COVID-19 vaccination had abnormal ECGs, and ST changes were present in 86% of patients. Oster et al. reported that 72% of patients with myocarditis after mRNA COVID-19 vaccination, including adolescent males, had abnormal ECGs. Although acute myocarditis may lead to sinus tachycardia with non-specific ST-segment and T wave abnormalities on ECG (20), the sensitivity of ECGs for the detection of myocarditis is inadequate (47%) (20). However, based on the results of the present and previous studies, ECG may be useful for the diagnosis of myocarditis after mRNA COVID-19 vaccination. Because ECG abnormalities may not be present at the time of presentation (as in Patient 3 in this study), repeated ECGs should be performed when myocarditis is suspected after mRNA COVID-19 vaccination.

The serum troponin I level was elevated in all patients except one in this study. An increased serum troponin I level was observed at the initial presentation in all patients except Patient 3. Troponin I is a highly specific marker of myocardial damage, with high specificity but limited sensitivity for the diagnosis of myocarditis (18). Das et al. (15) reported an increased troponin I level in all 25 of their patients with myocarditis after mRNA COVID-19 vaccination. Osler et al. (17) reported elevated troponin levels in 98% of patients with myocarditis after mRNA COVID-19 vaccination. Therefore, the serum troponin I level may be useful for the diagnosis of myocarditis after mRNA COVID-19 vaccination. The serum troponin I level increases 3–6 h after the onset of myocardial damage, peaks at 12–24 h, and normalizes within 2–3 days. Therefore, the diagnostic value of the serum troponin I level varies according to the time since the onset of myocarditis. In Patient 3 in this study, the serum troponin I level was not elevated at the time of presentation, but reached the upper limit of normal 3 days later. Interestingly, Patient 3 also had a normal ECG at initial examination, but exhibited ST elevation after 4 h; this may be explained by the short interval between the onset of myocarditis and presentation.

There were several limitations to this study. First, it was a retrospective, single-center case series. Thus, the results cannot be generalized. Second, the frequency of myocarditis may have been overestimated because our hospital is easily accessible for patients with myocarditis. In addition, none of the patients developed myocarditis after receiving vaccines other than BNT162b2; therefore, the frequency of myocarditis after other vaccine types could not be determined. Cardiac MRI was performed only in one patient in our study. Recent studies have suggested the usefulness of cardiac MRI in the diagnosis of myocarditis (21). Thus, we may had missed patients with myocarditis showing mild forms of atypical manifestations. We selected patients with myocarditis within 1 week after vaccination, because we presumed that myocarditis would appear within 1 week after COVID vaccination, based on previous reports (12). There is a possibility that we overlooked patients with myocarditis longer than 1 week after vaccination. On the other hand, we could not have completely excluded patients with myocarditis due to causes other than COVID vaccination, as we did not perform systematic viral screening.

## Conclusion

We report five cases of myocarditis after mRNA COVID-19 vaccination. All patients were males and the median interval from vaccination to the onset of myocarditis was 3 days. All patients had chest pain, as well as ST and serum troponin I elevation, suggesting the usefulness of these parameters for diagnosing myocarditis after mRNA COVID-19 vaccination. Although myocarditis after mRNA COVID-19 vaccination appears to be mild, further studies are required to confirm this.

## Data availability statement

The original contributions presented in the study are included in the article/supplementary material, further inquiries can be directed to the corresponding author.

## Ethics statement

Ethical review and approval was not required for the study on human participants in accordance with the local legislation and institutional requirements. Written informed consent from the participants' legal guardian/next of kin was not required to participate in this study in accordance with the national legislation and the institutional requirements. Written informed consent was not obtained from the minor(s)' legal guardian/next of kin for the publication of any potentially identifiable images or data included in this article.

## Author contributions

HMu contributed to the study design, interpretation of date, and was a major contributor in writing the manuscript. YZ, KS, HMi, NS, and NN contributed to the acquisition and analysis of date from the patients. HMo, HI, and AO revised the manuscript critically for important intellectual content. All authors read and approved the final manuscript as submitted and agree to be accountable for all aspects of the work.

## Conflict of interest

The authors declare that the research was conducted in the absence of any commercial or financial relationships that could be construed as a potential conflict of interest.

## Publisher's note

All claims expressed in this article are solely those of the authors and do not necessarily represent those of their affiliated organizations, or those of the publisher, the editors and the reviewers. Any product that may be evaluated in this article, or claim that may be made by its manufacturer, is not guaranteed or endorsed by the publisher.
